# GenoPheno: cataloging large-scale phenotypic and next-generation sequencing data within human datasets

**DOI:** 10.1093/bib/bbaa033

**Published:** 2020-04-06

**Authors:** Alba Gutiérrez-Sacristán, Carlos De Niz, Cartik Kothari, Sek Won Kong, Kenneth D Mandl, Paul Avillach

**Affiliations:** 1 Department of Biomedical Informatics, Harvard Medical School; 2 Department of Biomedical Informatics, Harvard Medical School; Computational Health Informatics Program, Boston Children's Hospital

**Keywords:** Large-scale datasets, phenotypic data, next-generation sequencing data, biobanks, catalog, precision medicine

## Abstract

Precision medicine promises to revolutionize treatment, shifting therapeutic approaches from the classical one-size-fits-all to those more tailored to the patient’s individual genomic profile, lifestyle and environmental exposures. Yet, to advance precision medicine’s main objective—ensuring the optimum diagnosis, treatment and prognosis for each individual—investigators need access to large-scale clinical and genomic data repositories. Despite the vast proliferation of these datasets, locating and obtaining access to many remains a challenge. We sought to provide an overview of available patient-level datasets that contain both genotypic data, obtained by next-generation sequencing, and phenotypic data—and to create a dynamic, online catalog for consultation, contribution and revision by the research community. Datasets included in this review conform to six specific inclusion parameters that are: (i) contain data from more than 500 human subjects; (ii) contain both genotypic and phenotypic data from the same subjects; (iii) include whole genome sequencing or whole exome sequencing data; (iv) include at least 100 recorded phenotypic variables per subject; (v) accessible through a website or collaboration with investigators and (vi) make access information available in English. Using these criteria, we identified 30 datasets, reviewed them and provided results in the release version of a catalog, which is publicly available through a dynamic Web application and on GitHub. Users can review as well as contribute new datasets for inclusion (Web: https://avillachlab.shinyapps.io/genophenocatalog/; GitHub: https://github.com/hms-dbmi/GenoPheno-CatalogShiny).

**Definitions**

- **Biobanks:** Repositories of biological specimens intended for clinical research. Biobanks store information about every specimen, such as the demographics and clinical symptoms or diagnoses of the subject from which it was sourced. In some cases, genotypic data associated with the specimen are also available.
- **Data descriptors:** Data about the data also known as metadata. In this manuscript, data descriptors specifically refer to: name of dataset, country where data were collected, disease focus, number of subjects with genomic and clinical data, number of phenotypic variables per patient, phenotypic data type, number of samples, molecular data type and the link to the website (when applicable).
- **Dataset:** A collection of clinical and genomic variables recorded for a cohort of subjects participating in a research study.
- **Repository:** Where datasets are stored, organized and distributed (e.g. dbGaP, EGA).

## Data repositories for precision medicine

The goal of precision medicine [1–4] is to identify targeted therapies for patients and to understand the etiology and progression of disease through the holistic and longitudinal analysis of patients’ genomic variant profiles, environmental exposures, lifestyle and digital health data. We can more quickly achieve these objectives by integrating multiple layers of patient information, such as clinical and genetic data; providing better access to integrated patient information for authorized clinical and biomedical researchers; and more richly characterizing patient conditions by including a significant number of variables extracted from adequately sized patient cohorts.

Large-scale human clinical and genetic datasets are proliferating worldwide. Between 2014 and 2019, the number of studies in the NCBI database of Genotypes and Phenotypes (dbGaP) [5] increased from 483 to more than 1300. Nevertheless, many studies are limited by small sample size, the lack of ancestral diversity and the lack of detailed phenotypic information associated with genomic variant data. Initiatives such as the All Of Us Research Program [6] and the UK Biobank [7, 8] seek to overcome these limitations. These datasets contain electronic health record (EHR) data, questionnaires and physical measurements along with genomic data of the same patients with either whole-genome or whole-exome sequencing (WGS/WES) data. Other initiatives, such as TopMed [9], enrich studies with detailed phenotypic information by adding the power of WGS and performing joint variant calling analysis across the different studies. Datasets like these speed up the pace of medical discovery while ensuring reproducibility.

Despite their potential value, many datasets lack data descriptors—also known as ‘metadata’—such as patient cohort size, number of recorded biosamples, sequencing platforms and access requirements or costs. In a few cases, descriptors are either scattered across the Internet or contained only in related manuscripts [10, 11]. In other cases, when available, descriptors are more often projections of long-term objectives rather than indicative of current status. This is unfortunate, because the information could improve dataset discoverability and usage as well as help researchers to determine a given dataset’s relevance to a particular research objective prior to devoting resources for requesting access to content.

We see, therefore, a need for a centralized, up to date, international catalog, designed to assist the global scientific community by listing patient-level genotypic and phenotypic datasets that might be useful for health-related research. We propose a dynamic and open source catalog that allows the scientific community to update existing datasets and submit new ones for inclusion, following peer review.

**Figure 1:**
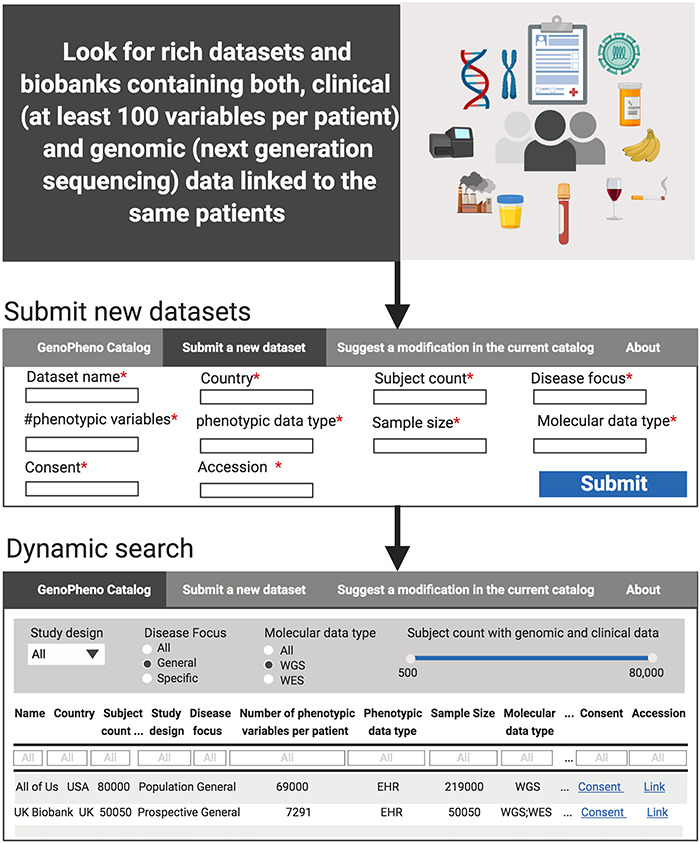
Curation and usage of the catalog. After locating rich datasets containing both phenotypic and genomic data from the same patients, the descriptive information is extracted from each dataset, submitted through the Shiny App form and displayed in an interactive Shiny app, where users can run dynamic searches.

Here, we provide a sample of datasets, containing both patient-level clinical and genomic data. In addition, we created a dynamic, online catalog for the biomedical research community to consult, add contributions and review (Figure 1). Our catalog is solely a listing of datasets along with the descriptors of those datasets provided by the investigators. To be clear, it is not a repository of clinical or genetic data. Our contribution is in gathering descriptive information—often widely scattered—about these datasets, making the information of the datasets readily discoverable. Importantly, we allow contributions to the catalog from the research community.

## Searching available datasets

We identify datasets with patient-level clinical and genomic data from a variety of sources. One source was dbGaP [12], a repository that hosts 1359 patient-level datasets with genotypic and phenotypic information. Each dataset in dbGaP contains multiple data descriptors, such as disease focus and study design, number of subjects, selected publications related to the study, applicable consent groups, molecular data types, marker sets, sequencing technology and assays, and computed ancestry. All the dataset descriptors are publicly available and downloadable from the dbGaP website. Biobanks are another source of phenotype and genotype data. In addition to storing biological samples, some biobanks also store corresponding phenotypic and genomic data. We performed a Web search for biobanks using the keywords: next-generation sequencing (NGS), patient-level, phenotype, data, biobank and biobank catalog. Most biobanks describe their disease focus, year of creation and number of subjects.

## Inclusion criteria

We defined six criteria for dataset inclusion in this review.

(i) **Including at least 500 human subjects.**
(ii) **Containing both genotypic and phenotypic data for every patient**. This allows comparative studies such as Phenome Wide Association Studies (PheWAS) [13] to be performed within and between individual patients and subpopulations. PheWAS evaluates the association of a genotype to a specific or multiple phenotype as well as the associations between different phenotypes. It is useful to explore the pleiotropic effect of genes—particularly when a genotype can be associated with multiple phenotypes [14]—and to examine comorbidities.
(iii) **Genomic data content includes WGS or WES data**. NGS, a parallel genome-processing tool with high-throughput capabilities, enables the exploration of the entire genome, rather than a very limited set of single nucleotide polymorphisms. This tool has become broadly available, in part due to significant reductions in cost over time [15]. NGS is now generally affordable, enabling high-coverage genome mapping for millions of individuals and variant discovery for targeted disease treatment [16–19]. These capabilities make NGS particularly useful and desirable for clinical applications.
(iv) **At least 100 clinical variables are recorded for each dataset**. Many clinical datasets include extensive demographic and consent information, i.e. recording variables for demographic data such as age, ethnicity, sex, weight, height, body mass index and various consent parameters for patient data use. In fact, some datasets cumulatively contain more than 50 of these demographic and consent variables. When seeking to establish genotype-to-phenotype associations in standard biomedical research, however, such demographic and consent data are only of limited utility. Thus, we arbitrarily chose a minimum of 100 clinical variables recorded for each patient. This bound is intended to keep the studies that aim to thoroughly collect phenotypes, rather than just minimal metadata appended to the genomic data.
(v) **Accessible through a website or through collaboration with investigators.**
(vi) **Information detailing access to the dataset is available in English**. Many biobanks throughout the world contain descriptions in a local language. Since English is the *lingua franca* of the worldwide biomedical research community, instructions for access must follow suit in order to enable global usage of a dataset and its utility to the Precision Medicine Initiative. Non-English datasets were not included in our review and catalog.

**Figure 2:**
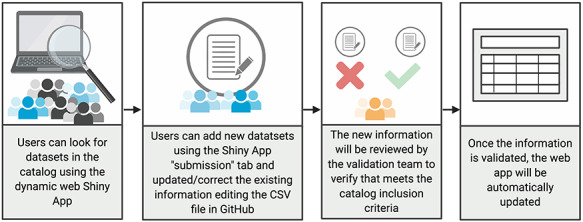
New submission and validation process. Users first search for the information available in the current version of the catalog. Those interested in updating or adding new information can edit the CSV file in GitHub. New information will be reviewed and the accepted changes in the CSV file in GitHub will automatically update the Web Shiny app.

Though the catalog described in this paper is by no means exhaustive, it is intended to be a living resource, accessible via a dynamic Web-user interface through which the scientific community can submit details about datasets for peer-review and acceptance. The catalog itself does not contain patient data. Instead, the catalog details only the characteristics of participating datasets and how to access the data within them. The methodology used for the creation of this catalog is depicted below (Figure 2).

## Datasets that meet the inclusion criteria

At the outset of our work, there were no search tools (website search and dbGaP Web) available for us to directly select datasets, based on the six inclusion criteria described. Therefore, we had to: (i) identify potential datasets; (ii) extract the dataset descriptors; (iii) carry out manual curation and (iv) filter based on the inclusion criteria. We followed these steps for both dbGaP and biobanks that we found on the Internet.

We also included datasets of which we had prior knowledge that satisfied the inclusion criteria.

### dbGaP datasets

Of the 1359 datasets present in dbGaP, 41% contain NGS data, 41% have more than 500 subjects and 11% have more than 100 clinical variables. Only eight datasets satisfied all the inclusion criteria (Genome-Wide Association Study of Amyotrophic Lateral Sclerosis, Genotype-Tissue Expression (GTEx) [20], UIC ACE Exome Sequencing Analysis [21], Sequencing of Targeted Genomic Regions Associated with Smoking [22], Genetic Study of Atherosclerosis Risk (GeneSTAR) [23], Genetics of Left Ventricular Hypertrophy (HyperGEN) [24] and WES in Tourette Disorder in Simplex Trios-TIC Genetics) [25].

For 216 datasets, dbGaP provides additional information about their relationship to each other. After the data descriptors for these related studies were combined, only 10 studies satisfied all the inclusion criteria and were included in this review. For example, there are three different datasets arising from the Genetic Epidemiology of Chronic Obstructive Pulmonary Disease (COPDGene) [26] study: phs000179.v6.p2, phs000296.v5.p2 and phs000765.v3.p2. Individually, none of these datasets would have satisfied the criteria for inclusion in our catalog. Study accession number phs000179.v6.p2 includes 341 clinical variables for more than 500 patients, but the molecular data type is not provided. Dataset with study accession number phs000296.v5.p2 includes NGS data but only for 289 patients and 20 clinical variables, and the dataset with study accession number phs000765.v3.p2 includes NGS data for 9991 patients but only 7 clinical variables. However, these three datasets are interrelated. Specifically, phs000179.v6.p2 is the ‘parent study’, according to dbGaP. Datasets phs000296.v5.p2 and phs000765.v3.p2 are considered ‘substudies’. By combining these three datasets into a single entity, we enabled COPDGene to collectively meet our inclusion criteria. Using this relationship-based strategy, we were able to add 10 datasets to the review: Framingham Cohort [27], Genetic Epidemiology of COPD (COPDGene), Women’s Health Initiative (WHI) [28], Atherosclerosis Risk in Communities (ARIC) Cohort [29], the Jackson Heart Study (JHS) [30], Cardiovascular Health Study (CHS) Cohort [31], Orofacial Pain: Prospective Evaluation and Risk Assessment (OPPERA) [32], T2D-GENES: San Antonio Mexican American Family Studies (SAMAFS), NINDS Parkinson’s Disease [33, 34] and Pediatric Cardiac Genomics Consortium (PCGC) Study [35].

For the remaining 1143 datasets, information about the relationship between datasets is not provided by dbGaP. For example, two different datasets in dbGaP arose from the Multi-Ethnic Study of Atherosclerosis (MESA) [36] (phs001416.v1.p1 and phs000209.v13.p3). Independently, neither met our inclusion criteria; phs001416.v1.p1 only contains 16 clinical variables, while phs000209.v13.p3, although having 22 147 clinical variables, does not provide the type of molecular data required for inclusion. At the same time, the specific Web entry in dbGap for phs001416.v1.p1 states that phenotypic data for MESA study participants are available within phs00209. However, the latter is not identified as a substudy, barring the collective inclusion of both databases in our review.

To circumvent the problem, we obtained the relationship information for these studies from the NIH Trans-Omics for Precision Medicine (TOPMed) program (https://www.nhlbiwgs.org) [9]. For background, TOPMed seeks to integrate different layers of information, including WGS and clinical data, from more than 80 distinct studies that focus on four main groups of disorders (heart, lung, blood and sleep disorders). The WGS data in TOPMed’s datasets are characterized by joint genotype calling, an efficient genotype call detection across thousands of samples to improve accuracy in variant discovery and increasing sensitivity in regions with low coverage [37, 38]. This means that although TOPMed’s studies were not large individually, their deployment of joint variant calling produced a robust multisample Variant-Calling Format file, consistent for the entire population.

We used the information available for TOPMed Freeze 5b, which contains 32 study accession numbers (https://www.nhlbiwgs.org/topmed-whole-genome-sequencing-project-freeze-5b-phases-1-and-2), alongside their corresponding parent study accession numbers, when available. With this additional information, we added five datasets to the review: MESA, Genetics of Lipid Lowering Drugs and Diet Network (GOLDN) [39], Study of Adiposity in Samoans (SAS) [40], the Cleveland Family Study (CFS) [41] and Genetic Epidemiology Network of Arteriopathy (GENOA) [42].

We manually reviewed each study to confirm information accuracy. We found that sample counts were not categorized by molecular data type. For example, Genome-Wide Association Study of Amyotrophic Lateral Sclerosis (phs000101.v5.p1) contains 15 480 genomic samples extracted from 15 480 subjects. However, NGS data are provided for only 409. Therefore, this dataset was discarded, as it was considered too small to meet our criteria.

Similarly, patient counts alone did not capture the true number of patients for whom both clinical and genomic data were recorded. This was the case with HyperGEN, where 2104 subjects had associated phenotypic data, but only 1773 also had recorded genotypic data. When faced with this discrepancy, we chose the smaller number of subjects (containing both clinical and genomic data) as the cohort size. We manually checked and updated these values for all of the datasets. Missing descriptors for these datasets had to be manually obtained from manuscripts.

Overall, 22 datasets from dbGaP (https://www.ncbi.nlm.nih.gov/projects/gapsolr/facets.html) satisfied the six criteria for inclusion as of 18 November 2019.

### Biobanks obtained after website exploration

The Web search for biobanks did not yield a listing of actual biobanks. Instead, we obtained links to interconnected Web pages, all of which had to be thoroughly reviewed to find necessary information. In general, data descriptors for each biobank, if available, were scattered and inconsistent. As a consequence, searching for biobanks by just using the inclusion criteria yielded only one result, the UK Biobank.

We then searched for existing catalogs of biobanks and found a few listings, based on different classification criteria. One listing was the catalog of European Biobanks directory [43] with 608 biobanks (as of 22 November 2019), hosted by the Biobanking and BioMolecular Resources Research Infrastructure-European Research Infrastructure Consortium [44]. This catalog focuses on biobanks that strictly collect biological samples. However, it does not include dataset descriptors that would suit the criteria of this review, such as number of subjects, number of phenotypic variables or NGS data.

We manually searched for the data descriptors of more than 20 biobanks, starting with the largest (in terms of participant number) that had websites in English. We discovered the ‘All of Us’ Biobank [6], with one million participants, and the FINNGEN Biobank or China Kadoorie Biobank, with approximately 500 000 participants. After filtering, based on our inclusion criteria, only two biobanks were included in our review (All of Us and UK Biobank [7]). Listed in Supplementary Table 1, the excluded biobanks were either missing NGS data or did not have information about the number of available NGS samples. An example is the FinnGen Biobank with 809 phenotypic variables per patient and more than 300 000 samples, but no available WGS or WES data.

### Datasets identified prior to this review

Given our research focus on autism and undiagnosed diseases, we had known a priori about four datasets that also met the six inclusion criteria: the Simons Simplex Collection (SSC) [45], the Undiagnosed Diseases Network (UDN) [46], the Boston Children PrecisionLink Biobank [47] and the Genomics Research and Innovation Network (GRIN) [48]. The SSC dataset contains more than 5459 clinical variables and 4784 genomic samples, obtained from 2392 subjects. The UDN dataset contains 3965 clinical variables and 462 genomic samples from 1042 subjects. The Boston Children PrecisionLink Biobank dataset encompasses 73 077 clinical variables and 500 WES samples from 500 subjects. Finally, the GRIN dataset incorporates 19 649 clinical variables and 500 WES genomic samples from 500 subjects.

### Description of the selected datasets

This review catalogs 30 datasets that, as of 20 November 2019, met our six inclusion criteria (Table 1, Supplementary Figure 1). To build this resource at what we view as an acceptable standard, we also undertook a manual curation process. We emphasize that the current version is not an exhaustive list. Rather, this catalog is meant to be an online resource and starting point for investigators who are searching for datasets. The script to perform automatic dataset selection, as well as the information manually added, is available at the GitHub repository: https://github.com/hms-dbmi/GenoPheno-CatalogShiny/blob/master/catalogGenerator.R

**Table 1:** Subset of six of 18 columns from the online catalog version 20 November 2019. The table is sorted in decreasing order, based upon the number of subjects with associated genomic and clinical information. The latest, complete version of the table with additional information (e.g. country, genotype markerset, patients’ age and ancestry, consent groups and accession links to the datasets) is available at https://avillachlab.shinyapps.io/genophenocatalog/

Name	Subject count with genomic and clinical data	Study design	Number of phenotypic variables per patient	Phenotypic data type	Number of genomic samples
All of Us [6]	80 000	Population-based cohort	48 682	EHR; questionnaires; physical measurements	219 000
UK Biobank [7]	50 050	Prospective study	7291	EHR; questionnaires; physical measurements; lifestyle	50 050
COPDGene[26]	10 371	Case-control	341	Patient/disease registries	10 776
WHI [28]	10 000	Prospective longitudinal cohort	6208	Patient/disease registries	20 010
PCGC study [35]	9444	Prospective observational cohort	435	Patient/disease registries	8411
MESA [36]	4875	Prospective longitudinal cohort	22 147	Patient/disease registries	4875
FHS [27]	4154	Prospective longitudinal cohort	61 988	Patient/disease registries	8326
CHS [31]	3622	Prospective longitudinal cohort	14 718	Patient/disease registries	3622
ARIC [29]	3612	Prospective longitudinal cohort	18 704	Patient/disease registries	6667
The JHS [30]	3406	Prospective longitudinal cohort	4690	Patient/disease registries	6812
Sequencing of targeted genomic regions associated with smoking [22]	2969	Case-control	131	Patient/disease registries	6196
OPPERA prospective cohort study of first-onset TMJD [32]	2866	Prospective longitudinal cohort	1061	Patient/disease registries	2866
SSC [45]	2392	Family/Twin/Trios	5459	Questionnaires	4784
HyperGEN [24]	1773	Family/Twin/Trios	164	Patient/disease registries	1776
GeneSTAR [23]	1636	Prospective longitudinal cohort	157	Patient/disease registries	3420
OPPERA baseline case-control study of chronic TMJD [70]	1608	Case-control	1079	Patient/disease registries	1608
SAS [40]	1222	Cross-sectional	182	Patient/disease registries	1232
GENOA [42]	1143	Prospective longitudinal cohort; Family/Twin/Trios	1118	Patient/disease registries	1143
WES in Tourette Disorder in Simplex Trios-TIC Genetics [25]	1104	Family/Twin/Trios	106	Patient/disease registries	1104
T2D-GENES Project 2: SAMAFS; substudy 2: WGS in pedigrees	1048	Family/Twin/Trios	272	Patient/disease registries	1651
UDN [46]	1042	Prospective longitudinal cohort	3965	HPO terms by clinical experts	462
NHGRI ClinSeq	1001	Case set	177	Patient /disease registries	1001
The CFS [41]	994	Prospective longitudinal cohort	2339	Patient/disease registries	1988
GTEx [20]	980	Cross-sectional	269	Patient/disease registries	3155
GOLDN [39]	898	Prospective longitudinal cohort	123	Patient/disease registries	1859
NHLBI GO-ESP: Heart Cohorts Exome Sequencing Project (ARIC)	843	Case-control	127	Patient/disease registries	1686
NINDS Parkinson’s Disease [33,34]	618	Case-control	113	Patient/disease registries	1223
UIC ACE Exome Sequencing Analysis [21]	523	Family/Twin/Trios	194	Patient/disease registries	1066
Boston Children Precision Link Biobank [47]	500	Prospective longitudinal cohort	73 077	EHR	500
GRIN [48]	500	Pediatric Network	19 649	EHR	500

For each dataset, we provided detailed information, including dataset name, country where the research took place, the number of participants with both genomic and clinical data recorded, study design (e.g. cohort, prospective and longitudinal), disease focus (e.g. general or disease-specific), number of phenotypic variables, phenotypic data type (e.g. EHRs, questionnaires and clinical notes), total number of DNA samples with genotype data, molecular data type (e.g. WGS and WES), genotype markerset (e.g. genotyping microarrays or on a sequencing technology basis), patients’ age and ancestry, consent (e.g. biomedical, disease-specific), accession link to the dataset or contact information to obtain data access, links to the originating clinical and genomic study, and PubMED identifier of the main publication(s) describing the dataset, if any. Table 1 shows 9 of 18 columns that are available in the online version of the catalog (https://avillachlab.shinyapps.io/genophenocatalog/). This table is publicly available on GitHub (https://github.com/hms-dbmi/GenoPheno-CatalogShiny/blob/master/csv/tableData.csv).

## Perspectives on included datasets

***Size:*** The datasets in the catalog were sorted by the number of subjects for which both phenotypic and genomic data had been recorded. In terms of subject cohort size, the All of Us dataset [6] was the largest, containing 80 000 subjects. The smallest was the Boston Children PrecisionLink Biobank [47], which contains data from 500 patients. Of note: the phenotypes of patients broadly consented and enrolled in the Boston Children PrecisionLink Biobank were the best-characterized among all the datasets in the catalog, recording up to 73 077 phenotypic variables.

***Source of clinical data*:** We could distinguish many sources of clinical data, with registry data and EHRs being the most common [49–52]. Each carried different advantages and limitations. For example, registries recorded whether or not a participant possessed a clinical variable. In the COPDGene [53] study, specifically, patients answered the question: ‘Have you ever had asthma?’ with any one of four different options: ‘yes’, ‘no’, ‘I don’t know’ or ‘missing data.’ In an EHR system, in contrast, data were in the form of codes that had been used for billing purposes. An advantage of EHR data is that it contains records of all visits to an institution. However, the record of any particular visit might lack a report of primary symptoms or comorbidities that support a diagnosis. From this record, an investigator can only ascertain the presence or absence of the diagnosis. Typically, the absence of the diagnostic code is translated as the patient not having that diagnosis. But just as likely, it may be that the symptoms or main comorbidities were not a primary element of that particular visit. This passive, negative assumption is different from the clear ‘no’ that is actively recorded in registry datasets. As a way around the limitation, recent studies have demonstrated the usefulness analyzing EHR clinical data to discover or confirm outcome correlations, find subcategories of disease and identify adverse drug effects [54, 55]. The EHR data structure allows a researcher, after the data has been collected, to view the full ICD hierarchy and so assess whether or not a patient did, in fact, have the disease or not. This desire for a much broader spectrum of all potential diagnoses led the Boston Children PrecisionLink Biobank to collect 73 077 clinical variables and All of Us to gather 69 000, based on ICD classifications. In contrast, the COPDGene collected merely 341 clinical variables and the WES in Tourette Disorder in Simplex Trios-TIC Genetics [25] used only 106, hindering subsequent analysis.

***Genomic samples*:** All of Us [6] and UK Biobank [7] had the most subjects with associated genomic information, 219 000 and 50 050 samples, respectively. NGS capabilities and continual technological improvement have enabled investigators to explore the human genome in detail and use that knowledge to treat complex diseases. With a significant reduction in cost in the last decade, NGS has enabled the mapping of genomes for millions of individuals, leading to the generation of extremely large volumes of data [56, 57], which is useful for clinical applications. An alternative is WES data that cover only the protein-coding regions of the genome (approximately 1.5%), but still allows deep coverage. We have focused exclusively on the subset of subjects that provided associated WGS or WES data.

***Disease focus*:** While there were four more generalized registries (All of Us, the UK Biobank, Boston Children PrecisionLink Biobank and GRIN), most of the datasets included in this review had focused on one disease. Of the specific-disease datasets, six targeted cardiovascular diseases [MESA, Framingham Heart Study (FHS), Cardiovascular Heart Study, the JHS, ARIC and the Heart Cohorts Exome Sequencing Project].

***Study type*:** The datasets originated from five categories of studies: case-control, prospective-cohort, cross-sectional, family and population-based. Of note, population-based studies were not necessarily disease-specific but always localized to a specific geographical area. An example is the FHS [27], which recorded clinical variables of patients with cardiovascular disorders in the town of Framingham, MA. Similarly, the UK Biobank [7] recorded clinical and genomic variables for more than 500 000 adult patients in the UK.

Five of the datasets were family studies, with the SSC [45] being the largest. SSC was originally created to enhance the discovery of rare and *de novo* variants in autism spectrum disorders (ASD). It encompasses 2700 families in which a child has ASD, unaffected biological parents and, in some cases, one or more unaffected siblings. Serving as the basis for more than 1400 publications, SSC is a good example of collaboration between several institutions.

***Subject age:*** We were able determine the subjects’ ages for 27 of the 30 datasets located. In general, age was not included in the webpage of the study. Rather, this information was more likely to appear in manuscripts derived from the data. Similarly, we could only ascertain subject ancestry for 23 of 30 total studies. Most datasets included in this review contain adults (>18 years old). Only four datasets focus mainly on children: SSC [45], Undiagnosed Disease Network (UDN), the CFS and the Boston Children PrecisionLink Biobank [47]. While Boston Children’s Hospital treats mainly children, 20% of patients are older than 18 years. The latter are often patients with very rare conditions that continued to come to the hospital for follow-up into adulthood in order to preserve a continuum of case. They represent a special case, as their parents consented for them as children and the patients themselves had to reconsent, upon request, after reaching 18 years of age [58].

***Consent:*** Information about consent is also part of this review, and the consent type or the link to the consent information is available in the ‘Consent’ column of the online version of the catalog. For some cases, such as the Boston Children PrecisionLink Biobank and the SSC, the link to the research consent document for data use agreement, signed by the patient, is available. For other cases, such as UK Biobank, GRIN and All of US, the catalog contains a link to a website with consent-related information. For the rest of the datasets, hosted in dbGaP, the description of the different content types is displayed. Importantly, the same study can present several patient consent groups that might impact subsequent studies. For example, the COPDGene study contains 290 patients who signed disease-specific (COPD and smoking) consents and 10 328 patients who signed health/medical biomedical consents. This means that, when performing research unrelated to COPD and smoking, data from the disease-consented 290 patients cannot be used. In contrast, all the patient data can be used for future smoking research.

## Access

Typically, the following steps are required to obtain access to a dataset: (i) the database evaluation based on the dataset descriptors available; (ii) the identification of the requirements to access the data; (iii) writing a proposal and (iv) Data User Agreement compliance and IRB approval. To enable dataset accessibility, the online version of the catalog contains an accession link or contact information. These will bring the user to a website or document with detailed guidance to prepare data access request. Twenty seven out of the 30 datasets are publicly available, while three of them (Boston Children PrecisionLink Biobank, UDN and GRIN) are only accessible via principal investigator collaboration.

## Contributing to this catalog

Based on this review, we created the GenoPheno catalog. It represents a dynamic and open-source dataset for the scientific community to use. Specifically, scientists can add new information and/or correct existing data. They can submit a new dataset by going to the webpage and clicking on the ‘submit a new dataset’ tab (https://avillachlab.shinyapps.io/genophenocatalog/). Contributors can correct existing information via the GitHub repository, using the CSV file containing available catalog information (https://github.com/hms-dbmi/GenoPheno-CatalogShiny/blob/master/csv/tableData.csv) (Figure 2). Currently, a validation team of four investigators, the developers or the catalog, will periodically review the entered data. Eventually, other validators will join the review.

For consideration, new submissions should include the following: name of the dataset, country where data was collected, disease focus, number of subjects with genomic and clinical data, number of phenotypic variables per patient, phenotypic data type, number of samples, molecular data type, and the link to the website (when applicable) or directions and/or contact information for the investigators to whom correspondence should be addressed. Following validation, the CSV file will be updated and used to update the website.

## Challenges

Overall, the main challenge in performing this review was the procurement of information. Data were scattered and lacked consistency in its descriptors (e.g. self-reported ancestry/race/ethnicity). In many cases, the relationships between multiple, derivative datasets were not always evident. This created many difficulties in accurately determining patient cohort sizes, sample specifications, availability of WGS data and other parameters that would aid in making these datasets discoverable and accessible.

For instance, to have a clear idea about how derived information from each dataset can be used, it would be helpful to include the license information for the datasets as an additional dataset descriptor in the catalog. However, since some sources lack license information, and this is addressed in the user agreement when requesting access to the dataset, GenoPheno provides the direct link to request data access and the applicable consent(s).

We manually curated and reviewed the datasets and made our work available to others through the GenoPheno catalog. In this way, we believe that we have facilitated the discoverability, accessibility and reusability of these datasets. Our work is synchronous with ongoing standardization initiatives, such as the Findability, Accessibility, Interoperability and Reusability guiding principles [59] that serve to guide data producers and publishers in their quest to properly manage and steward data. Their relevance to precision medicine has been recognized by the biomedical research community [60–62].

## Limitations

We found three main limitations when building this catalog: (i) the lack of data descriptors; (ii) biobank websites not written in English and (iii) difficulty in distinguishing biobanks with samples from those that also contained processed information.

A large number of datasets worldwide have descriptive information available only in the vernacular language. The lack of descriptive information in English restricts the use of these datasets to a geographical region or a specific user community. More importantly, data from these nationally—and possibly ethnically diverse patient cohorts—are rendered unavailable for analysis to the larger global research community. For these reasons, we inserted in the inclusion criterion a requirement that access instructions be available in English and excluded non-English datasets from this review.

Shared ancestry has been identified as a confounding factor in association studies such as genome wide association studies carried out on ethnically homogeneous patient cohorts [63], leading to the identification of false positives. On the other hand, association studies performed on patient cohorts from diverse ancestries (multi-ethnic studies) have identified associations between several novel genetic variants and phenotypes not commonly observed in patient populations with the most commonly studied European ancestry [64–67]. To further this type of discovery, we encourage data managers worldwide to provide access instructions and descriptors in English.

We also considered the European Genome-phenome Archive (EGA) [68] repository that contains datasets from 2400 studies. Of these, 348 datasets are also hosted on dbGaP. Data descriptors are not easily accessible on EGA nor are they specified. Where available, an independent query had to be executed on each study to extract the metadata. Due to these limitations, EGA datasets were not included in this review.

The time-intensive nature of compiling accurate descriptive information for the included datasets narrowed the scope of this review to 30 datasets. However, the limitation was addressed by releasing this information in a dynamic catalog and encouraging community participation in the review and update of the datasets, as well as in the submission of new datasets for inclusion.

We have not delved into the interoperable aspects of the datasets in this review. The wide heterogeneity between the clinical and genomic data variables captured in the various datasets in GenoPheno was a significant impediment to dataset interoperability. Major challenges in interoperability include the harmonization of the variables across the diverse datasets, the batch effects between studies and differences in protocols. To achieve interoperability, we suggest identifying biological associations in one study cohort and validating them in another.

The recorded clinical variables differed from study to study. This introduced complications in the development of analyses that utilize clinical data from different studies. Therefore, it will be necessary to identify variables that are shared across studies, especially variables that record more than patient demographic information. Harmonization of the clinical variables shared across studies is necessary because different studies will typically record the value of these variables differently, using different units for quantitative measurements or different scales for ordinal and categorical measurements. The harmonization of clinical variables involved painstaking human curation. As an example, the harmonization of a set of 43 clinical variables (e.g. ‘presence or absence of carotid plaque’, ‘body height at baseline’ and ‘body weight at baseline’) for over 230 000 patients across 17 studies took 2 years for a team of data scientists at the TOPMed Data Coordination Center [69].

## Conclusions

This review summarizes 30 patient-level datasets integrating both NGS genomic and phenotypic data to facilitate the discovery and access of these data to advance precision medicine research. Additionally, we present this information in the form of a dynamic, open-source catalog, GenoPheno, which can be accessed online by users who can review as well as contribute new datasets for inclusion.

## Supplementary Material

Supplementary_Table_And_Figure_bbaa033

## Notes

Key Points

- Datasets containing patient phenotypic and genomic (NGS) data are crucial to the advancement of precision medicine.
- Availability of dataset descriptors prior to requesting data access saves researchers time and effort, allowing them to focus on data analysis that is relevant to their work.
- This manual review of 30 datasets containing patient genotypic and phenotypic data, although not an exhaustive listing, is meant to launch a new attempt at standardization and better access for clinical and biomedical research communities.
- The scientific community is invited and encouraged to contribute and update the current content of this review, available as a dynamic online GenoPheno catalog.

## Notes

**Alba Gutiérrez-Sacristán** is a postdoctoral research fellow in the Department of Biomedical Informatics (DBMI) at the Harvard Medical School. Her research focuses on analyzing autism comorbidities and developing R software for data analysis.

## Notes

**Carlos De Niz** is a Postdoctoral Research Fellow in the DBMI at the Harvard Medical School. His research focuses on genomic analysis, cloud computing and studying the effect of accumulated mutations in autism patients.

## Notes

**Cartik Kothari** is a Data Scientist in the DBMI at the Harvard Medical School. His research focuses on analyzing clinical data related to patients diagnosed with rare genetic diseases.

## Notes

**Sek Won Kong** is an Assistant Professor in the Computational Health Informatics Program (CHIP) at Boston Children’s Hospital. His research focuses on precision medicine, translational genomics, clinical whole genome sequencing and autism spectrum disorder.

## Notes

**Kenneth D. Mandl** is the Director of the Computational Health Informatics Program at Boston Children's Hospital, and the Donald A.B. Lindberg Professor of Pediatrics and Biomedical Informatics at the Harvard Medical School.

## Notes

**Paul Avillach** is an Assistant Professor of biomedical informatics at the Harvard Medical School and the Principle and Co-Investigator on large projects within DBMI. He develops novel methods for data integration that encompass biological observations.
