# Supplementary material for: GenoPheno: cataloging large-scale phenotypic and next-generation sequencing data within human datasets
Source: Brief Bioinform. 2020 Apr 6;22(1):55–65. doi: 10.1093/bib/bbaa033 (PMC7820848; doi:10.1093/bib/bbaa033)
Supplement: Supplementary_Table_And_Figure_bbaa033 [file supplementary_table_and_figure_bbaa033.docx]

**Supplementary Table 1**. **Biobanks reviewed but not included in the catalog.**

This table includes the biobank name, the website (when available) and the reasons for exclusion. The table is sorted alphabetically by biobank name.

| **Biobank** | **Website** | **Exclusion reason** |
| --- | --- | --- |
| Australian Prostate Cancer BioResource | <https://www.apcbioresource.org.au/> | Lack of descriptors |
| BancoADN | <http://www.bancoadn.org/en> | Lack of descriptors |
| Biobank Graz |  | Lack of descriptors |
| Biobank Japan |  | Lack of descriptors |
| Canadian Partnership for Tomorrow Project Biobank |  | Lack of descriptors |
| Canadian Tumour Repository Network | <https://www.ctrnet.ca/> | Lack of descriptors |
| China Kadoorie Biobank | <http://www.ckbiobank.org/site/> | No NGS data available; only GWAS |
| Danish National Genome Center | [http://www.genomedenmark.dk](http://www.genomedenmark.dk/english/) | Lack of descriptors (number of subjects and number of phenotypic variables per subject, and sample size) |
| EuroBioBank network | <http://www.eurobiobank.org/> | Lack of descriptors |
| FinnGen | <https://www.finngen.fi/en/> | No NGS data available; only GWAS genotype and imputation |
| H3Africa | <https://h3africa.org/> | No enough number of subjects with NGS and phenotypic data (324 subjects). |
| Icelandic Healthcare Database (IHD) / deCODE | <https://www.decode.com/> | Lack of descriptors (number of subjects and number of phenotypic variables per subject, and sample size) |
| kConFab | [http://www.kconfab.org](http://www.kconfab.org/) | Lack of descriptors |
| Marshfield Biobank | <https://marshfieldresearch.org/irdl/lab-services/bio-banking> | Lack of descriptors |
| Mayo Clinic Biobank | <https://www.mayo.edu/research/centers-programs/mayo-clinic-biobank/overview> | Lack of descriptors |
| NeuroBioBank | <https://neurobiobank.nih.gov> | Lack of descriptors |
| Qatar Biobank | <https://www.qatarbiobank.org.qa/> | Lack of descriptors |
| Shanghai Zhangjiang Biobank | <http://www.shbiobank.com/> | Lack of descriptors |
| Victorian Cancer Biobank | <https://viccancerbiobank.org.au/> | Lack of descriptors |
| Wales Cancer Bank | [https://www.walescancerbank.com](https://www.walescancerbank.com/) | Lack of descriptors |

**
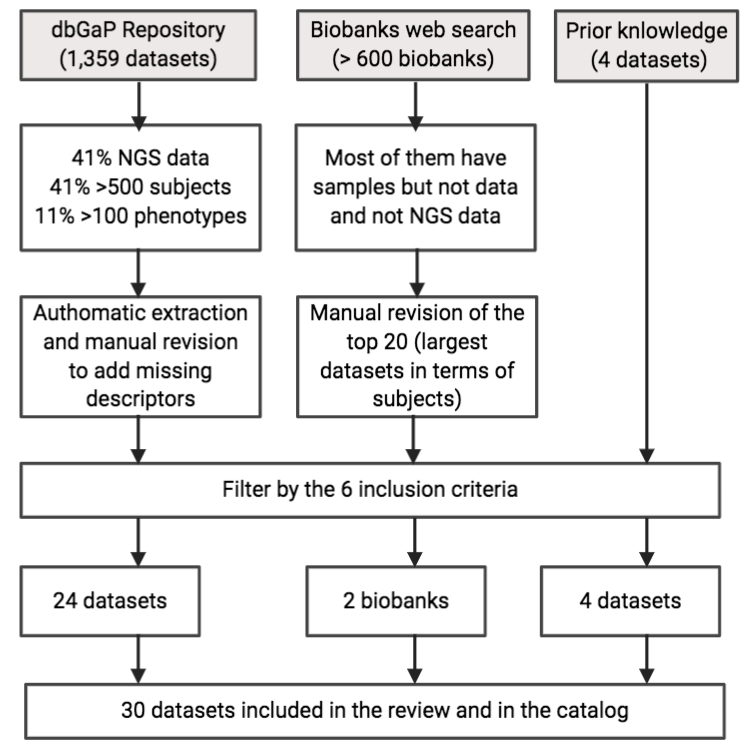
**

**Supplementary Figure 1. General workflow of the steps followed in this review.**

We began with the source of datasets, followed by the review of the descriptors, filtering by the inclusion criteria, resulting in a total of 30 datasets.
